# Gender differences in the association between body mass index and health-related quality of life among adults:a cross-sectional study in Shandong, China

**DOI:** 10.1186/s12889-019-7351-7

**Published:** 2019-07-31

**Authors:** Jiao Zhang, Lingzhong Xu, Jiajia Li, Long Sun, Wenzhe Qin, Gan Ding, Qian Wang, Jing Zhu, Zihang Yu, Su Xie, Chengchao Zhou

**Affiliations:** 1 School of Public Health, Shandong University, Jinan, 250012 China; 2 NHC Key Laboratory of Health Economics and Policy Research, Shandong University, Jinan, 250012 China; 3 Center for Health Economics Experiment and Public Policy Research, Shandong University, Jinan, 250012 China; 4 Collaborative Innovation Center of Social Risks Governance in Health, School of Public health, Fudan University, Shanghai, 200032 China

**Keywords:** Body mass index, Health-related quality of life, Gender, Underweight, Obesity

## Abstract

**Background:**

This study aims to assess the association between body mass index (BMI) and health-related quality of life (HRQOL), and to further explore gender differences in BMI-HRQOL association among adults.

**Methods:**

We used data from the fifth Health Service Survey of Shandong Province, which was part of China’s National Health Service Survey (NHSS), a total of 27,257 adults aged 18 and over were interviewed. The HRQOL was measured using the EuroQOL-5 Dimensions (EQ-5D) instrument. One-way ANOVA and Post hoc tests were used to compare EQ-5D utility values and visual analogue scale (VAS) scores between BMI categories. Tobit regression models were used to identify the association between BMI and HRQOL for male and female separately after controlling for influential confounders, and to assess gender differences on the relationship between BMI and HRQOL.

**Results:**

The prevalence of underweight in men and women were 3.2 and 5.3%, respectively, while the prevalence of overweight/obesity in men and women were 35.7 and 34.6%, respectively. Men had higher EQ-5D utility values and VAS scores than women. The mean EQ-5D utility value and VAS score was highest in obese men and normal-weight women, respectively. After controlling potential confounders, being underweight was significantly and negatively associated with lower HRQOL among adults. The relationship between obesity and gender was that in women obesity was negatively and significantly associated with HRQOL, whereas in men this association was positive but not statistically significant. Results of gender by BMI interaction in regression model showed that this difference between men and women in this respect was significant.

**Conclusions:**

The association between BMI and HRQOL differed by gender and the so-called “obesity-HRQOL paradox” phenomenon was verified in male adults*.* Gender difference should be considered when implementing targeted weight control programs and appropriate interventions to improve HRQOL.

## Background

A recent pooled analysis of 1698 population-based study with 19.2 million participants from 200 countries estimated that global age-standardized mean body mass index (BMI) was in an increasing trend for both men and women from 1975 to 2014, which will become a serious public health concern if post-2000 trends continue [1]. Some previous research showed that high BMI (overweight or obese) was an important risk factor for a variety of chronic diseases [2–5], and that may result heavy health and economic burden, such as increased mortality and morbidity and higher annual medical care cost [6–9]. In China, the prevalence of high BMI (overweight and obesity) has been increasing and is fast approaching epidemic proportions [10]. The Report on Nutrition and Chronic Diseases of Chinese Residents (2015) revealed that the prevalence of overweight and obesity among adults aged 18 years were 30.1 and 11.9%, increased by 7.3 and 4.8 percentage compared with that in 2002 separately. Whether for young people or adults, the growth rate of high BMI in China was higher than that in the most developed countries [11]. Simultaneously, much lower BMI (underweight) remained prevalent and was also a serious problem in China [12]. There was evidence that much lower BMI (underweight) was associated with physical, functional, and psychological impairment and increased risk of chronic disease and mortality [13, 14]. Therefore, both much higher and lower BMI are likely to impair the overall health of population.

Health-related quality of life (HRQOL) has been recognized as a valid health indicator with the realization that biomedically oriented measures of a population’s well-being, such as mortality and morbidity rates, to provide a partial picture of public health needs and prevention outcomes [15, 16]. Some studies have examined the association between BMI and HRQOL in general population or specific population [17–19].

However, the impact of BMI on HRQOL may vary by gender. Some studies have showed that much higher or lower BMI was negatively associated with HRQOL in both men and women, including decreasing physical or emotional well-being or both [18, 19], while some study indicated that at higher BMI values, men reported higher HRQOL than women; at lower BMI values, HRQOL was lower in men than women [20], and another study reported that the association between obesity and lower HRQOL remained significant for women, but not for men [21]. In the current sociocultural context, people still value physical attractiveness and thinness, particularly in women, which may contribute in higher risk of experiencing low self-esteem, negative body image, and depression for women than men. By contrast, some people regard obesity a protective factor, especially for men or patients with chronic diseases. As a few studies have reported, high BMI (overweight or obesity) was associated with slightly better HRQOL as compared to those with normal weight [22–24]. Excess weight is paradoxically associated with a decreased risk of adverse outcomes, and the ‘obesity paradox’ has been proposed as a label for above unexpected results [25, 26]. Another study conducted by Yanbo Zhu [27] showed that those with the class I obese (25–29.9 kg/m^2^) had better HRQOL in both the physical and the mental domains than those with normal weight, especially in the mental well-being, which extended the “obesity paradox” to HRQOL outcomes. Therefore, there is no consistent recognition of the association between high or low BMI and HRQOL.

Most studies assessing the association between BMI and HRQOL among Chinese adults focused on obesity group or weight in age-specific groups [28–30], or did not further explore the gender difference explicitly for the above association [12]. A better understanding of the mechanisms will help in the design of more targeted and appropriate interventions. However, the differences on BMI- HRQOL association between men and women are still inconclusive, and no similar in-depth investigations were conducted among adults from Shandong Province, where the total population ranks second in China. To fill the gap literature, the aims of our study are 1) to evaluate the association between BMI and HRQOL among adults sampled from Shandong Province, China, and 2) to further explore gender differences on association between BMI category and HRQOL among adults.

## Methods

### Data and sample

We used data from the fifth Health Service Survey of Shandong Province, which was part of China’s National Health Service Survey (NHSS), conducted in 2013. The NHSS is a national survey commissioned by the Nation Health and Family Planning Commission of China every 5 years since 1993, which used multistage stratified cluster sampling to select 94 of 2,859 counties from China’s 31 provinces and municipalities. The Shandong’s NHSS in 2013 covered total 17 prefectures across the province, and 20 counties, 100 towns were randomly selected through a multistage stratified cluster sampling method. In the first stage, 20 counties were selected from total 137 counties as the primary sampling units (PSUs) throughout the province. In every PSU, 10 villages were selected as the secondary sampling units (SSUs). In the last stage, 60 households were randomly selected from every SSU. Finally, a total of 12,010 households, consisting of 33,070 individuals, were included in the sample. After verbal informed consent was obtained from interviewees, a face-to-face interview was conducted individually for all members in every selected household using a structured household questionnaire. The sample population was, therefore, an excellent source of health and sociodemographic variables within the population of Shandong.

Given our focus on adult sample, we restricted our analysis to respondents aged 18 years or older, and 27,257 adult respondents were included in this study after excluding respondents whose BMI or HRQOL data were missing.

### Variables

#### Body mass index

Body mass index (BMI) was collected by physical measurement and was calculated as weight (kg) divided by the square of height (m^2^). BMI was classified based on WHO guidelines as follows: underweight was a BMI<18.50; normal weight was 18.5 ≤ BMI<25.0; overweight was 25.0 ≤ BMI<30.0; obese was BMI ≥ 30.0 [31]. Category “very obese” was combined into “obese” since only 105/27,257 (0.39%) population with BMI ≥ 35.0 kg/m^2^ in our study. Although BMI itself has been questioned as the optimal measurement to use for assessing obesity, other measures such as waist circumference, waist: hip ratio, and waist: height ratio, may be better. However, BMI remains the most commonly used, widely accepted, and practical measure of obesity in both children and adults, particularly for surveillance [32].

#### Heath-related quality of life

The Chinese version EQ-5D-3 L, was preselected as a measure in Shandong’s NHSS 2013. The EQ-5D-3 L was a generic, preference-based instrument comprising a health classification system with five dimensions (mobility, self-care, usual activities, pain/discomfort and anxiety/depression), each dimension with three response levels (no problem, some problems, extreme problems) and a visual analogue scale (VAS), which recorded the respondent’s perception of overall health status on a scale from 0 (indicating the worst imaginable health) to 100 (indicating the best imaginable health). The health classification system described a total of 243 health states, each of which was assigned a utility weight, range − 0.149 to 1, using a utility scoring function derived from Chinese general population using the time trade-off method [33]. Higher utility values represent higher HRQOL.

#### Other variables

Data on demographic characteristics, health behaviors and self-reported diseases/conditions were included in this study as follows: gender (Male, Female), age (continuous), area (Urban, Rural), education attainment (Primary school or below: illiteracy or primary school, Junior school, Senior school or above: senior school or technical school or secondary school or associate’ degree or bachelor’s degree and above), smoking status (Yes: daily smoker or casual smoker, No: nonsmoker) and drinking status (Have you had any alcohol in the past 12 months? Response: Yes/No), exercise times weekly (< 1: never or less than once, 1–5: 1–2 times or 3–5 times, > 6: 6 times and above), health examination (Have you had a physical examination in the past 12 months that does not include a medical examination? Response: Yes/No), number of chronic diseases (Have you been diagnosed with hypertension/diabetes/chronic obstructive pulmonary disease/cancer/periodontitis/other chronic diseases? Response for each item: Yes/No; The total number of answers to “yes” was divided into four categories: 0, 1, 2, > 3), family members with cancers (Are there cancer patients in your family? Response: Yes/No).

### Statistical analysis

Data were described using either means (standard deviations) or numbers (proportion), where appropriate. Gender differences in sociodemographic characteristics were analyzed using a *t*-test (for continuous variables) or *Chi-square* test (for categorical variables) as appropriate. One-way ANOVA and Post hoc tests were used to compare EQ-5D utility values and VAS scores between BMI category. Our dependent variable (EQ-5D utility value) was censored (− 0.149, 1) and our data were skewed, and many respondents were at the upper limit. Tobit regression model was employed in our study based on the above reasons, which was appropriate to evaluate factors related to HRQOL measured by the EQ-5D utility value as discussed in many studies [34–36]. Thus, Tobit regression models were undertaken for men and women separately to assess the association between BMI category and HRQOL after controlling for influential sociodemographic and health-related factors, with EQ-5D utility values as the dependent variable. Then, gender differences on the association between BMI category and HRQOL were examined by adding the gender by BMI interaction term into the regression model. All data were analyzed using SPSS 24.0 and STATA 14.0. *P*-values<0.05 were considered to be statistically significant.

## Results

### Respondents’ characteristics

Descriptive statistics for the sample are shown in Table 1. In a total of 27,257 adults, men and women accounted for 47.8 and 52.2% separately. Men had a slightly lower mean age and significantly higher mean BMI (*P*<0.001) and comprised a higher proportion of the population who were highly educated (*P*<0.001), smoker (*P*<0.001), drinker (*P*<0.001) than women. The proportion of men with one or more chronic diseases was 30.9%, slightly lower than that of women (*P*<0.01). The mean (SD) BMI of respondents were 24.02 ± 3.40 and 23.68 ± 3.51 for men and women, respectively. The prevalence of underweight in men and women were 3.2 and 5.3%, respectively, while the prevalence of overweight/obesity in men and women were 35.7 and 34.6%, respectively. The mean (SD) EQ-5D utility values and VAS scores in men were 0.95 ± 0.14 and 83.24 ± 15.03 respectively, both of which were higher than that in women (*P*<0.001).

**Table 1:** Sociodemographic and health-related conditions by gender

Variable	Total	Men	Women	*P*-value
	27257	13032 (47.8)	14225 (52.2)	
Age, mean ± SD	50.03 ± 16.04	49.83 ± 15.85	50.21 ± 16.2	0.036^a^
Area [n (%)]				<0.001^b^
Urban	18371 (67.4)	8646 (66.3)	9725 (68.4)	
Rural	8886 (32.6)	4386 (33.7)	4500 (31.6)	
Education attainment [n (%)]				<0.001^b^
Primary school or below	9740 (35.7)	3404 (26.1)	6336 (44.5)	
Junior school	9906 (36.3)	5357 (41.1)	4549 (32.0)	
Senior school or above	7611 (27.9)	4271 (32.8)	3340 (23.5)	
Smoking status [n (%)]				<0.001^b^
Yes	6216 (22.8)	5976 (45.9)	240 (1.7)	
No	21041 (77.2)	7056 (54.1)	13985 (98.3)	
Drinking status [n (%)]				<0.001^b^
Yes	7623 (28.0)	7136 (54.8)	487 (3.4)	
No	19634 (72.0)	5896 (45.2)	13738 (96.6)	
Exercise times weekly [n (%)]				0.227^b^
≥6	3501 (12.8)	1722 (13.2)	1779 (12.5)	
1–5	4050 (14.9)	1919 (14.7)	2131 (15.0)	
<1	19706 (72.3)	9391 (72.1)	10315 (72.5)	
Health examination [n (%)]				<0.001^b^
Yes	12581 (46.2)	5727 (43.9)	6854 (48.2)	
No	14676 (53.8)	7305 (56.1)	7371 (51.8)	
Number of chronic diseases [n (%)]				0.001^b^
0	18546 (68.0)	9005 (69.1)	9541 (67.1)	
1	5838 (21.4)	2739 (21.0)	3099 (21.8)	
2	2214 (8.1)	988 (7.6)	1226 (8.6)	
≥3	659 (2.4)	300 (2.3)	359 (2.5)	
Family members with cancers				0.640^b^
Yes	243 (0.9)	123 (0.9)	120 (0.8)	
No	27014 (99.1)	12859 (98.7)	14054 (98.8)	
BMI categories [n (%)]				<0.001^b^
Underweight	1173 (4.3)	417 (3.2)	756 (5.3)	
Normal weight	16516 (60.6)	7960 (61.1)	8556 (60.1)	
Overweight	8376 (30.7)	4068 (31.2)	4308 (30.3)	
Obese	1192 (4.4)	587 (4.5)	605 (4.3)	
BMI, mean ± SD	23.84 ± 3.46	24.01 ± 3.40	23.68 ± 3.51	<0.001^a^
EQ-5D, mean ± SD	0.95 ± 0.14	0.95 ± 0.14	0.94 ± 0.14	<0.001^a^
VAS, mean ± SD	82.56 ± 15.29	83.24 ± 15.03	81.94 ± 15.50	<0.001^a^

*SD* standard deviation, *BMI* body mass index, *EQ-5D* EuroQOL-5 Dimensions, *VAS* visual analogue scale, ^a^ *t*-test, ^b^ *Chi-square* test

### Comparison of HRQOL between BMI category

**Fig. 1:**
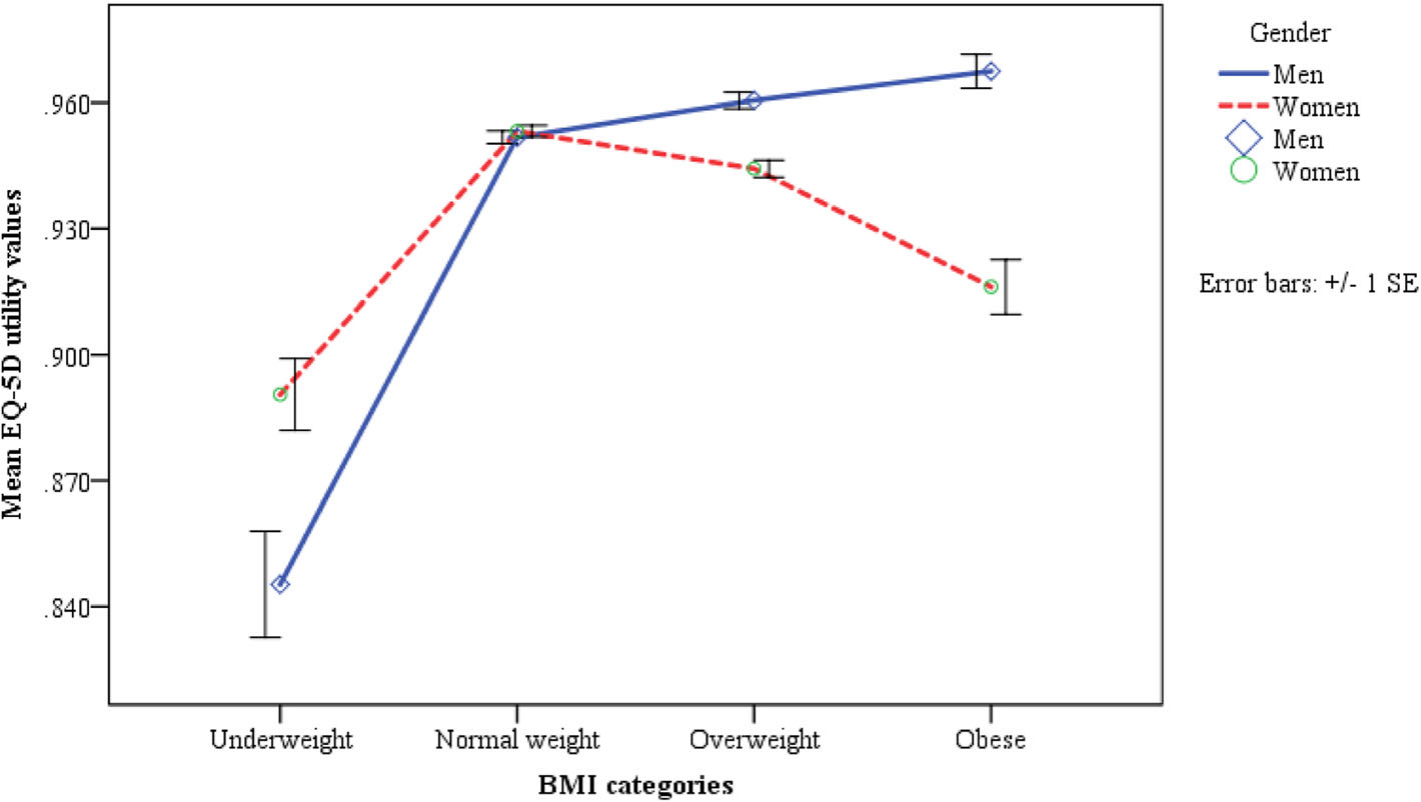
Mean EQ-5D utility values between BMI category by gender

Table 2 shows the results of comparing EQ-5D utility values and VAS scores between BMI category in men and women separately. There were gender differences in both mean EQ-5D utility values and VAS scores between four BMI categories. For men, post hoc *test* results showed that underweight group had lower HRQOL (P<0.001), while overweight (P<0.01) and obese groups (P<0.001) had significant higher HRQOL, compared with the normal weight group. For women, however, some different pattern was revealed. Women with both underweight (P<0.001) and overweight (P<0.01) or obesity (P<0.01) were likely to report lower HRQOL than those with normal weight. The mean EQ-5D utility value was highest in obese men and normal-weight women (See also in Fig. 1). Similar results were also found for men and women regarding VAS scores.

**Table 2:** Comparisons of EQ-5D utility values and VAS scores between BMI category

	Underweight	Normal weight	Overweight	Obese	*P*-value ^a^
*Men*					
EQ-5D utility values, mean ± SD	0.85 ± 0.26	0.95 ± 0.14	0.96 ± 0.12	0.97 ± 0.10	<0.001
VAS scores, mean ± SD	73.85 ± 21.06	83.33 ± 14.92	83.94 ± 14.22	84.06 ± 14.21	<0.001
*Women*					
EQ-5D utility values, mean ± SD	0.89 ± 0.24	0.95 ± 0.13	0.94 ± 0.13	0.92 ± 0.16	<0.001
VAS scores, mean ± SD	78.35 ± 19.41	83.01 ± 15.12	80.85 ± 15.20	79.29 ± 15.44	<0.001

*SD* standard deviation, ^a^ One-way ANOVA test for differences of BMI category

### Association between BMI and HRQOL and its gender differences

Table 3 summarizes Tobit regression models of HRQOL measured by EQ-5D. First, we presented separate regression models for both men and women (Table 3, Model I and Model II). Compared with the normal weight group, the underweight group reported significantly lower HRQOL in both men (β = − 0.148, *P*<0.001) and women (β = − 0.099, *P*<0.001). However, for overweight and obese group, a slightly different pattern was shown between men and women. Obesity had a significant negative effect on HRQOL in women (β = − 0.068, P<0.01, Model II), but not in men (Model I). More importantly, the difference between men and women in this respect was significant (β = 0.103, *P*<0.01, Model III).

**Table 3:** Tobit regression models for association between BMI category and HRQOL and its gender difference

Variable	Model I Men		Model II Women		Model III Interaction: Gender × BMI	
	Coefficient	95%CI	Coefficient	95%CI	Coefficient	95%CI
Age	−.011***	(−.012, −.010)	−.012***	(−.013, −.011)	−.011***	(−.012, −.011)
Urban (vs rural)	.055***	(.026, .083)	.045***	(.023, .071)	.048***	(.030, .066)
Education attainment (vs Primary school or below)						
Junior school	.102***	(.074, .130)	.055***	(.031, .079)	.077***	(.059, .095)
Senior school or above	.115***	(.081, .150)	.071***	(.039, .104)	.091***	(.068, .114)
Smokers	.026*	(.002, .049)	.014	(−.044, .073)	.025*	(.004, .045)
Drinkers	.096***	(.072, .119)	−.026	(−.072, .020)	.072***	(.052, .092)
Exercise times weekly (vs ≥ 6)						
1–5	−.092***	(−.136, −.048)	−.098***	(−.133, −.062)	−.095***	(−.123, −.068)
<1	−.140***	(−.177, −.103)	−.147***	(−.177, −.117)	−.143***	(−.166, −.120)
Health examination	−.063***	(−.087, −.039)	−.064***	(−.082, −.046)	−.064***	(−.079, −.049)
Number of chronic diseases (vs 0)						
1	−.186***	(−.213, −.159)	−.177***	(−.199, −.156)	−.181***	(−.198, −.164)
2	−.299***	(−.336, −.262)	−.271***	(−.299, −.243)	−.283***	(−.306, −.261)
≥ 3	−.385***	(−.442, −.327)	−.347***	(−.392, −.303)	−.365***	(−.400, −.329)
Family members with cancers	−.175***	(−.261, −.089)	−.208***	(−.283, −.132)	−.193***	(−.249, −.136)
BMI categories (vs Normal weight)						
Underweight	−.148***	(−.200, −.095)	−.099***	(−.137, −.061)	−.106***	(−.147, −.066)
Overweight	.023	(−.002, .049)	−.002	(−.021, .018)	−.004	(−.024, .017)
Obese	.032	(−.026, .090)	−.068**	(−.107, −.028)	−.074**	(−.116, −.032)
Men (vs Women)					−.047***	(−.069, −.025)
Gender × BMI						
Men × underweight					−.034	(−.097, .028)
Men × overweight					.026	(−.006, .057)
Men × obese					.103**	(.035, .171)
Constant	2.083***	(1.998, 2.168)	2.163***	(2.097, 2.229)	2.156***	(2.103, 2.208)
Respondents	13032		14225		27257	
*R* ^2^	.237		.285		.262	

*CI* confidence interval,* *P*<0.05, **P<0.01, ***P<0.001, Gender×BMI = interaction effect between gender and BMI category

## Discussion

Gender differences on association between BMI and HRQOL was investigated explicitly in our study. Overall, overweight and obese adults accounted for 30.7 and 4.4%, respectively, and there was a higher proportion of overweight/obese men than women. The highest mean EQ-5D utility value and VAS score was achieved by men of obese group and by women of normal weight group, respectively. After controlling potential confounders, gender had a significant effect in the association between BMI categories and HRQOL, especially for obese group.

A lot of attention was paid to dealing with obesity-HRQOL, and the relationship between underweight and HRQOL was likely to be ignored. Nevertheless, an obvious fact in our study was that being underweight was associated with the worst HRQOL and our findings were broadly consistent with those reported in many studies [12, 17, 23, 27, 37]. Previous studies have reported that underweight was associated with increased risk of excess mortality [38], poor cognition [39, 40], and poor self-rated health [41]. A previous study based on population in five cities of China also showed that there was more percentage of underweight persons (8.64%) than obesity (3.27%) in China [29]. In view of this, the neglected underweight problems need as much attention as obesity.

Generally, overweight and obesity were negatively associated with HRQOL, a surprising but interesting finding in our study was that obesity had a negative impact on women’ HRQOL after adjusting for other influential factors including chronic diseases, but not for men. A possible explanation about gender differences in obesity was that women might be more susceptible to distress to body weight or body image than men [42, 43]. Excessive dieting may result in lower HRQOL among obese women. It could also be explained by cultural perceptions about personal body weight and more discrimination against women with excess body weight in their work-related life and social roles [44]. Considering this, we performed another Tobit regression analysis to estimate the effects of the interactions of gender by BMI on HRQOL. As the results showed, women suffered from the negative effects of obesity on HRQOL even after adjusting for demographic, life style and chronic condition variables. All of those imply that we need to pay more attention on the overweight and obese females when improving HRQOL. Awareness to challenges facing those women can help health care providers work more effectively with them to develop different plans for losing weight, adopting healthier lifestyles.

The relationship between BMI and HRQOL in our study varied by gender, especially for population with obesity. Many studies have shown that the “obesity paradox”, a “paradoxical” decrease in morbidity and mortality with increasing BMI, existed in elderly or patients with chronic conditions [24, 45–47]. Recent studies extended the “obesity paradox” to HRQOL outcomes, suggesting that overweight and obesity may paradoxically correlate with higher HRQOL, called “obesity-HRQOL paradox” [22, 27, 48]. There has been some emerging evidence that the association between obesity and HRQOL may be stronger in women than men [29, 49, 50]. According to the available data, our finding is first to verify that this “obesity-HRQOL paradox” phenomenon appeared in Chinese male adults. Cultural and social atmosphere could well explain why obese men was positively associated with HRQOL. In Chinese traditional culture, being fat was not a symbol of unhealthiness because only the wealthier people can afford eating more and can put on more weight. A more recent Chinese saying described becoming fat during middle age as acquiring a good fortune [51]. Also, it may be a matter of special concept in China that some people regarded fat as “robust”, “affluent” state, especially for men [52]. Our results were also consistent with the results reported by Audureau et al. [53] that the significant “obesity-HRQOL paradox” were found in men for mentally oriented HRQOL domains. The increased amounts of nonfat and muscle mass, which are important contributors to fitness or overall health, might be another important concept in understanding this obesity paradox [54]. However, there is wide agreement that BMI is seriously flawed because it is unable to distinguish between lean body mass and fat mass, and BMI may underestimate the number of people for whom fatness impacts health [55]. Therefore, further studies based on other measures should be carried out to was needed to explain the phenomenon, but in this study, we confirmed that gender as a moderator had a significant effect on the relationship between BMI on HRQOL among adults. Accordingly, more targeted and appropriate interventions should be conducted, especially in women, to decrease the detrimental obesity effects associated with HRQOL. Furthermore, given that HRQOL was used as input for cost-effectiveness analyses, findings from our study are important for identifying population subgroups in which weight management programs are potentially most cost-effective.

The limitations of this study should be acknowledged. Firstly, this was a cross-sectional study which may result that the causality between BMI and HRQOL cannot be inferred despite the clear association. Secondly, our analyses were not weighted by the appropriate sampling weights. However, a series of quality and consistency tests including Myer’s Index (Myer’s index =2.99), Test of Goodness for Fit(χ^2^ = 12.59, P>0.05), DELTA No-similarity Coefficient (Δ = 0.0915) and GINI Concentration Ratio (GINI = 0.1079) showed that our survey data had high quality and the sample had a strong population representativeness of Shandong Province. Thirdly, we did not conduct more different subgroup analyses in this study, and will have a more explicit analysis in the follow-up study. In addition, the NHSS only collected the BMI data as a measure of obesity, we could not compare BMI with other measures, such as waist-to-hip ratio and visceral measurement, to explore more accurately and comprehensively gender difference in the association. However, there are also several strengths in this study. Firstly, the data we used were sampled from the general population of the Shandong’ NHSS 2013, which gave this analysis excellent power. More importantly, we confirmed that the gender differences on relationship of BMI and HRQOL by adding the interaction term of gender by BMI into regression model. Finally, our present study verified the “obesity paradox” to HRQOL outcomes in the male adults.

## Conclusion

In this population-based study, we found that the underweight group had the lowest HRQOL, which deserved more attention. The effect of obesity on HRQOL in adults differed by gender, and it is critical to develop effective gender-specific weight control programs. The counter-intuitive “obesity- HRQOL” phenomenon was verified in male adults. Further research based on other measures are needed to investigate the mechanisms that underpin the gendered nature of obesity prevalence.

## Data Availability

The datasets used in the current study are not publicly available due to the confidential policy but are available from the corresponding author on reasonable request.

## Abbreviations

BMI: Body Mass Index
CI: Confidence Interval.
EQ-5D: EuroQOL-5 Dimensions
HRQOL: Health-related Quality of Life;
NHSS: National Health Service Survey
PSU: Primary Sampling Unit
SD: Standard Deviation
SSU: Secondary Sampling Unit
VAS: Visual Analogue Scale
WHO: World Health Organization
